# Hyperbaric Oxygen Therapy in Surgical Wound Healing and Tissue Salvage: A Structured Narrative Review

**DOI:** 10.7759/cureus.105894

**Published:** 2026-03-26

**Authors:** José Emiliano González Flores, Aarón Gonzalez Espinosa, Manuel Esaú Tamayo-Gómez, Stephanie Johanna Chon Pineda, Dalia Azucena de Luna Vega, Patricia Gomez Roblero, Juan Hiram Marquez Moreno, Aline Gonzalez Martinez, Emilio Godínez Lazarini, Jeffrey Barragan Ortega, Diego Ruiz, Andrea Navalón Calzada

**Affiliations:** 1 School of Medicine and Health Sciences, Tecnológico de Monterrey - Campus Ciudad de Mexico, Mexico City, MEX; 2 Department of Public Health, Anáhuac University, Mexico City, MEX; 3 Department of General and Plastic Surgery, Universidad Nacional Autónoma de México (UNAM), Mexico City, MEX; 4 Department of General Surgery, Hospital General de Especialidades "Dr. Javier Buenfil Osorio", Campeche, MEX; 5 School of Medicine and Health Sciences, Tecnológico de Monterrey - Campus Ciudad de México, Mexico City, MEX; 6 Department of Investigation, Instituto Nacional de Ciencias Médicas y Nutrición Salvador Zubirán, Mexico City, MEX; 7 School of Medicine, Universidad Juárez Autónoma de Tabasco, Villahermosa, MEX; 8 School of Medicine, Universidad Autónoma de Guadalajara, Guadalajara, MEX; 9 Department of Internal Medicine, Hospital Central Norte de PEMEX, Mexico City, MEX; 10 Department of Surgery, Hospital Español, Mexico City, MEX

**Keywords:** angiogenesis, chronic wounds, diabetic foot ulcers, flap ischemia, graft survival, hyperbaric oxygen therapy, postoperative complications, reconstructive surgery, surgical wound healing, tissue salvage

## Abstract

Surgical wound complications remain a major source of postoperative morbidity, particularly in the setting of tissue hypoxia, ischemia, and impaired perfusion. Hyperbaric oxygen therapy (HBOT) has emerged as an adjunctive modality aimed at enhancing tissue oxygen delivery and optimizing wound healing dynamics across diverse surgical contexts. A structured narrative review of the literature was conducted using PubMed/MEDLINE to identify clinical studies, systematic reviews, and translational investigations evaluating the role of HBOT in surgical wound healing and tissue salvage. The search included articles published from January 2000 to February 2026. Evidence was synthesized descriptively, focusing on mechanistic pathways, clinical indications, reconstructive applications, and reported outcomes.

HBOT exerts therapeutic effects through multiple physiological mechanisms, including enhanced plasma-dissolved oxygen delivery, angiogenesis stimulation, fibroblast proliferation, collagen synthesis, and immunomodulation. Clinical evidence supports its use in chronic ischemic wounds, such as diabetic foot ulcers, venous leg ulcers, burn injuries, and radiation-induced tissue damage, demonstrating improved wound closure rates and reduced infection risk. In reconstructive surgery, HBOT has shown benefit in compromised grafts, ischemic flaps, traumatic soft-tissue injuries, and breast reconstruction salvage. Reported outcomes include enhanced tissue viability, reduced necrosis progression, improved graft integration, and decreased amputation rates in high-risk populations.

HBOT represents a biologically active adjunct capable of enhancing surgical wound healing and tissue salvage through multifactorial regenerative mechanisms. As its applications continue to expand into reconstructive and aesthetic surgical domains, HBOT is positioned to play an increasingly integrative role within multidisciplinary perioperative care and complex wound management strategies.

## Introduction and background

Background

Surgical wound healing represents a fundamental determinant of postoperative recovery and procedural success across all surgical disciplines. Despite substantial progress in operative techniques, biomaterials, and perioperative management, impaired wound healing continues to impose a significant clinical and economic burden worldwide. Postoperative wound complications, including infection, dehiscence, ischemia, and tissue necrosis, are associated with prolonged hospitalization, increased reintervention rates, and, in severe cases, limb or tissue loss [1,2].

Tissue oxygenation plays a central role in the orchestration of wound repair. Adequate oxygen supply is essential for fibroblast proliferation, collagen deposition, angiogenesis, epithelialization, and oxidative bacterial killing. Hypoxic wound environments disrupt these processes, resulting in delayed healing, chronic inflammation, and increased susceptibility to infection [3]. In patients with systemic comorbidities, such as diabetes mellitus or peripheral vascular disease, compromised microcirculation further exacerbates tissue hypoxia, amplifying the risk of nonhealing surgical wounds [2].

Hyperbaric oxygen therapy (HBOT) has emerged as an adjunctive modality aimed at reversing tissue hypoxia and restoring physiological healing mechanisms. By delivering 100% oxygen under supra-atmospheric pressure conditions, HBOT increases plasma-dissolved oxygen concentrations, facilitating diffusion into ischemic wound beds, independent of hemoglobin-bound transport [4]. This hyperoxic effect enhances angiogenesis, stimulates fibroblast function, promotes collagen matrix formation, and potentiates leukocyte-mediated antimicrobial activity, thereby accelerating wound repair processes.

Rationale

Over recent decades, the clinical indications for HBOT have expanded beyond decompression illness and radiation injury to encompass a wide spectrum of surgical wound pathologies. Strong evidence supports its adjunctive role in chronic diabetic foot ulcers, where hyperbaric oxygen has been associated with improved healing rates and reduced amputation risk [2,5]. Similarly, HBOT has demonstrated therapeutic benefit in venous ulcers, burn injuries, and radiation-induced soft tissue damage, reinforcing its utility in both acute and chronic wound settings [1,6].

In addition to chronic wound management, HBOT has gained relevance in surgical tissue salvage. Compromised grafts, ischemic flaps, traumatic soft-tissue defects, and necrotizing infections represent clinical scenarios in which rapid restoration of oxygen delivery may determine reconstructive success. Experimental and clinical data indicate that hyperbaric oxygen enhances flap viability, supports graft integration, mitigates ischemia-reperfusion injury, and reduces local inflammatory responses [4,7-9].

Furthermore, emerging molecular evidence suggests that HBOT modulates gene-expression pathways involved in angiogenesis, stem-cell mobilization, and inflammatory regulation. These regenerative mechanisms extend its therapeutic potential beyond conventional wound healing, positioning HBOT as a biologically active adjunct within multidisciplinary surgical care frameworks [3].

Objective

Given the growing integration of HBOT into contemporary surgical wound management protocols, a comprehensive evaluation of its mechanistic foundations and clinical applications is warranted. The objective of this narrative review is to synthesize current evidence regarding the physiological mechanisms through which HBOT enhances wound healing, examine its role across diverse surgical wound etiologies, and assess its effectiveness in tissue salvage scenarios, with an emphasis on clinically relevant outcomes and reconstructive implications.

## Review

Methods

A structured literature search was conducted to identify relevant publications addressing the role of HBOT in surgical wound healing and tissue salvage. The search was performed in the PubMed/MEDLINE database from January 2000 to February 2026, prioritizing English-language articles. Search terms were applied to titles, abstracts, and indexed keywords.

The search strategy incorporated both Medical Subject Headings (MeSH) and free-text keywords, combined using Boolean operators. The following search string was applied: (“hyperbaric oxygen therapy” OR “HBOT”) AND (“surgical wound” OR “wound healing” OR “tissue salvage” OR “flap” OR “graft” OR “burn” OR “ischemic ulcer” OR “radiation injury”).

To ensure comprehensive coverage of relevant literature, manual searches of reference lists from key review articles and clinically relevant studies were also performed. Titles and abstracts identified through the search strategy were screened for relevance, followed by full-text evaluation of potentially eligible studies. Screening and study selection were conducted manually by the authors. Priority was given to publications with direct relevance to surgical wound healing, reconstructive surgery, ischemic wound management, and tissue salvage.

Eligible publications included clinical studies, systematic reviews, meta-analyses, and high-quality observational reports evaluating the role of HBOT in surgical or reconstructive wound contexts. Studies were required to report clinically relevant outcomes, including wound closure rates, graft or flap survival, infection control, angiogenesis, tissue perfusion, or regenerative healing metrics.

Exclusion criteria comprised experimental animal studies lacking clear translational clinical correlation, conference abstracts without full-text availability, editorials, and publications addressing non-surgical indications of HBOT, such as decompression sickness or carbon monoxide poisoning.

For each eligible publication, relevant information was extracted, including study design, patient population characteristics, HBOT treatment parameters (pressure, duration, and number of treatment sessions), clinical indications, and reported clinical outcomes. Extracted data were synthesized qualitatively to identify common mechanistic pathways, therapeutic indications, and reported clinical benefits associated with HBOT in surgical wound management.

Given the heterogeneity in study designs, patient populations, treatment protocols, and outcome reporting, findings were synthesized through a descriptive narrative framework rather than quantitative meta-analysis. This approach is consistent with the methodological flexibility inherent to structured narrative reviews, aimed at integrating mechanistic and clinical evidence across diverse surgical contexts.

Study Selection

**Figure 1:**
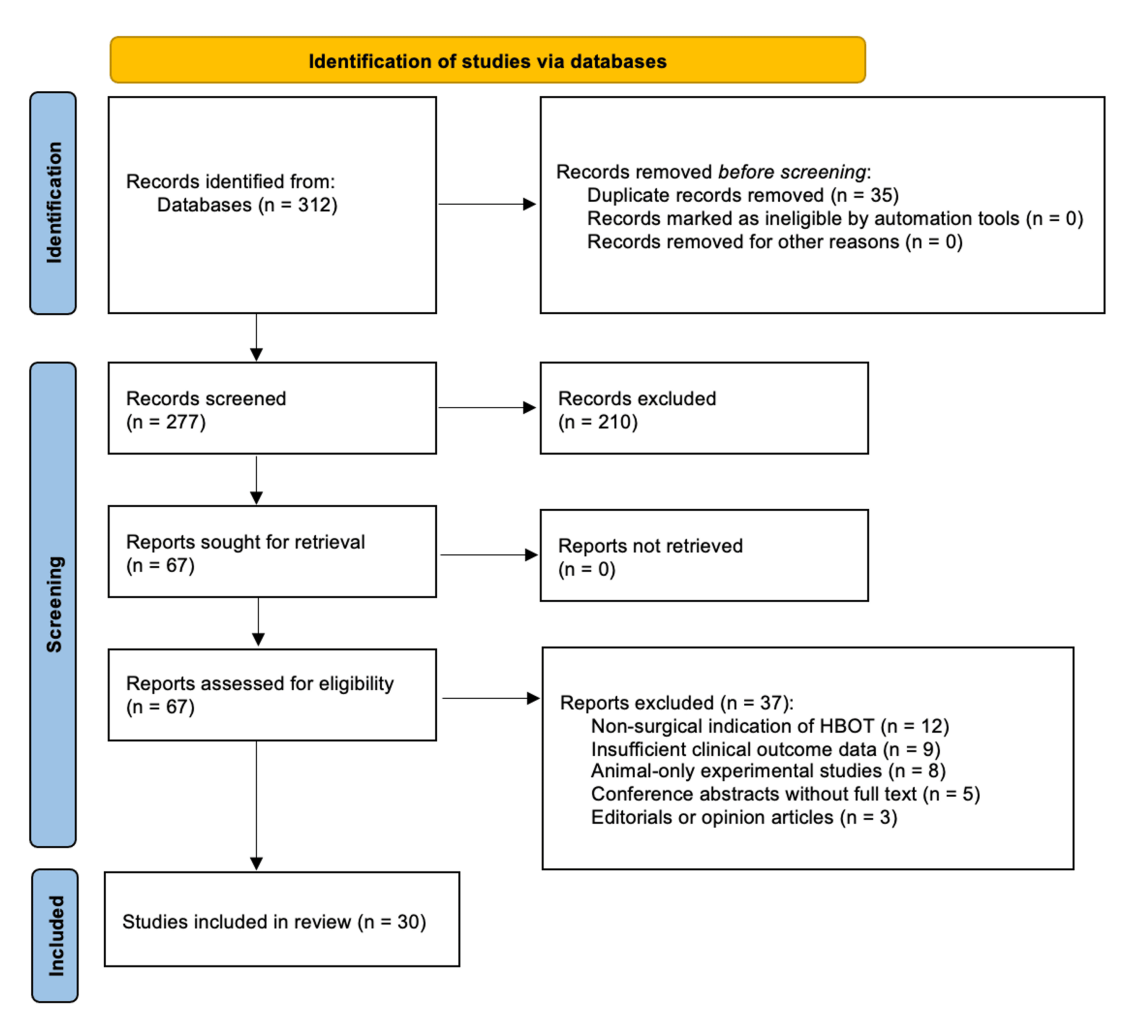
Study selection process for the literature review PRISMA-based flow diagram illustrating the process of identification, screening, eligibility assessment, and inclusion of studies evaluating the role of hyperbaric oxygen therapy (HBOT) in surgical wound healing and tissue salvage. A total of 312 records were identified through database searches. After the removal of 35 duplicate records, 277 articles underwent title and abstract screening, of which 210 were excluded. Sixty-seven full-text articles were assessed for eligibility, and 37 were excluded based on predefined criteria, including non-surgical indications of HBOT, insufficient clinical outcome data, animal-only experimental studies without clinical correlation, conference abstracts lacking full text, and editorial or opinion articles. Ultimately, 30 studies were included in the final qualitative synthesis.

The search strategy identified 312 records through database searches. After the removal of 35 duplicate records, 277 articles remained for title and abstract screening. Of these, 210 were excluded based on relevance. Subsequently, 67 full-text articles were assessed for eligibility, and 37 were excluded according to the predefined criteria. Ultimately, 30 studies were included in the final qualitative synthesis (Figure 1).

Although 30 studies met the inclusion criteria and were incorporated into the qualitative synthesis, only studies reporting sufficiently detailed clinical and methodological data were included in the comparative tables. Consequently, Tables 1-2 summarize 17 studies that provided complete outcome data, suitable for structured comparison, while the remaining studies were incorporated into the narrative synthesis.

**Table 1:** Clinical evidence of HBOT in chronic and ischemic surgical wounds Summary of key clinical studies evaluating the role of adjunctive HBOT in the management of chronic and ischemic surgical wounds. Included publications encompass systematic reviews, meta-analyses, cohort studies, and comparative clinical investigations, addressing indications such as diabetic foot ulcers, venous leg ulcers, burn injuries, radiation-induced soft tissue damage, and nonhealing postoperative wounds. Reported outcomes include wound closure rates, epithelialization time, infection control, graft integration, and amputation risk reduction, highlighting the therapeutic impact of hyperbaric oxygen across diverse wound etiologies. HBOT: hyperbaric oxygen therapy; DFU: diabetic foot ulcer; VLU: venous leg ulcer; ATA: atmospheres absolute

Author (Year)	Study Design	Indication	Sample	HBOT Protocol	Key Outcomes	Conclusion
Sharma et al. (2021) [2]	Systematic Review & Meta-analysis	Diabetic Foot Ulcers	14 studies (12 RCTs + 2 CCTs)	Variable	↑ Complete healing, ↓ major amputation	Adjunct HBOT may improve DFU outcomes
Zhang et al. (2022) [5]	Systematic Review & Meta-analysis	Diabetic foot ulcers	20 RCTs (1263 patients)	Variable	↑ Healing rate, ↓ healing time, ↓ major amputation	HBOT may improve DFU outcomes
Hu et al. (2025) [11]	Network Meta-analysis	Diabetic foot ulcers	99 RCTs (7,356 patients)	Variable	↓ Amputation risk vs standard care	HBOT shows benefit among adjunct therapies
Keohane et al. (2023) [6]	Systematic Review	Venous leg ulcers	6 studies	Variable	↓ Ulcer area	Limited evidence for HBOT in VLU
Weitgasser et al. (2021) [14]	Narrative Review	Burns	-	Variable	Improved wound healing mechanisms	Evidence insufficient for routine use
Teguh et al. (2021) [17]	Cohort Study	Nonhealing wounds	248 patients	2.4 ATA	81% healing or near healing	Effective adjunct therapy
Elsharnoby et al. (2025) [13]	Comparative Study	Resistant venous leg ulcers	33 ulcers	20-40 sessions	↑ Healing rate, ↓ closure time	Effective adjunct in refractory ulcers

**Table 2:** Clinical evidence of hyperbaric oxygen therapy in surgical tissue salvage and reconstructive applications Summary of clinical studies evaluating the role of adjunctive HBOT in surgical tissue salvage and reconstructive applications. Included evidence encompasses flap ischemia, graft compromise, traumatic soft-tissue injuries, breast reconstruction complications, aesthetic surgery outcomes, and complex postoperative wounds. Reported endpoints include graft integration, flap viability, necrosis reduction, infection control, and overall reconstructive success following HBOT. HBOT: hyperbaric oxygen therapy; ATA: atmospheres absolute; NSM: nipple-sparing mastectomy; BEE: bladder exstrophy-epispadias

Author (Year)	Study Design	Surgical Context	Sample Size	HBOT Protocol	Key Outcomes	Conclusion
Marra et al. (2024) [7]	Observational Study	Lower extremity trauma reconstruction	57 patients	2.5 ATA, 80-90 min sessions (~25 sessions)	Improved graft integration, ↓ necrosis	Adjunct HBOT enhances reconstructive stability
Seyhan and Öksüz (2022) [9]	Preclinical Experimental Study	Chronic wound model	46 rats	2.4 ATA, 90 min sessions (7 days)	↑ Fibroblasts, collagen, angiogenesis, ↓ wound area	HBOT accelerates wound healing when combined with PRP
Oley et al. (2022) [16]	Case Series	Fingertip flap salvage	18 patients	2.0 ATA	↑ Flap viability, ↓ necrosis	HBOT may improve outcomes in compromised flaps
Idris et al. (2024) [24]	Systematic Review	NSM complications after breast reconstruction	7 studies	Variable	↓ Ischemia/necrosis, ↑ flap salvage	HBOT may be a beneficial adjunct in post-mastectomy reconstruction
Nasr et al. (2023) [23]	Retrospective Study	Threatened flaps after nipple-sparing mastectomy	17 patients (25 breasts)	2.0 ATA, 90-min dives once or twice daily	88% flap salvage, low reoperation rate	HBOT may improve the salvage of threatened NSM flaps
Mortada et al. (2025) [21]	Systematic Review & Meta-analysis	Aesthetic surgery postoperative recovery	11 studies (734 patients)	2.0-3.0 ATA, 45-120 min sessions	↓ Healing time, ↓ complications, ↑ patient satisfaction	HBOT may improve postoperative recovery in aesthetic surgery
González Flores et al. (2026) [22]	Narrative Review	Multispecialty surgical wound healing	-	Variable	Mechanistic pathways and clinical evidence synthesis	HBOT supports tissue salvage across surgical specialties
Teguh et al. (2021) [17]	Prospective Cohort Study	Chronic nonhealing wounds	248 patients	2.4 ATA, 75 min sessions (~48 sessions)	81% complete/near-complete healing	HBOT improves healing in chronic wounds
Zaman et al. (2025) [18]	Retrospective Cohort Study	Diabetic foot graft reconstruction	45 patients	2.4 ATA, 120 min daily	↑ Graft retention, ↓ healing time	HBOT may improve graft survival in diabetic foot reconstruction
Hanna (2021) [25]	Case Series	Complex reoperative urologic reconstruction (BEE)	11 patients	1.5-2.0 ATA, 20 pre-op + 5-10 post-op sessions	Successful reconstruction, ↓ wound complications	HBOT may enhance healing in scarred reconstructive fields

Central body

Mechanistic Basis of HBOT in Wound Healing

HBOT exerts its therapeutic effects primarily through the reversal of tissue hypoxia, a central pathophysiological factor underlying impaired surgical wound healing. By administering 100% oxygen at supra-atmospheric pressures, typically between 2.0 and 2.5 atmospheres absolute, HBOT markedly increases the amount of oxygen dissolved in plasma, independent of hemoglobin-bound transport. This elevated oxygen tension enhances diffusion gradients, allowing oxygen delivery to ischemic wound beds, where microvascular perfusion is compromised [3,8].

At the cellular level, oxygen availability directly influences fibroblast proliferation and collagen synthesis, both of which are essential for structural wound repair. Hyperoxia stimulates fibroblast replication, promotes extracellular matrix deposition, and accelerates the transition from inflammatory to proliferative healing phases. Experimental models have demonstrated significantly enhanced wound tensile strength and faster epithelialization in hyperbaric environments compared with normoxic controls [4,9].

Angiogenesis represents another critical mechanism through which HBOT facilitates wound healing. Sustained hyperoxia induces vascular endothelial growth factor (VEGF) expression and promotes capillary budding within hypoxic tissues. This neovascularization improves long-term tissue perfusion, creating a self-sustaining oxygen delivery system, even after therapy cessation. Biomolecular studies further reveal upregulation of hypoxia-inducible factors and stem cell mobilization pathways, reinforcing the regenerative potential of HBOT beyond acute oxygen delivery [3].

In addition to promoting tissue regeneration, HBOT exerts potent antimicrobial effects. Oxygen-dependent leukocyte oxidative killing mechanisms, particularly neutrophil-mediated reactive oxygen species (ROS) production, are significantly enhanced in hyperoxic conditions. This effect is especially relevant in contaminated or infected surgical wounds, where impaired host immunity contributes to persistent bacterial colonization. Hyperbaric oxygen has demonstrated bacteriostatic and bactericidal activity against anaerobic pathogens and enhances antibiotic penetration into ischemic tissues [8].

The anti-inflammatory properties of HBOT also play a crucial role in optimizing wound healing. Hyperoxia modulates cytokine expression, reducing pro-inflammatory mediators while promoting anti-inflammatory signaling pathways. This immunomodulatory effect helps mitigate ischemia-reperfusion injury, a phenomenon frequently encountered in surgical flaps and grafts following revascularization [7].

**Figure 2:**
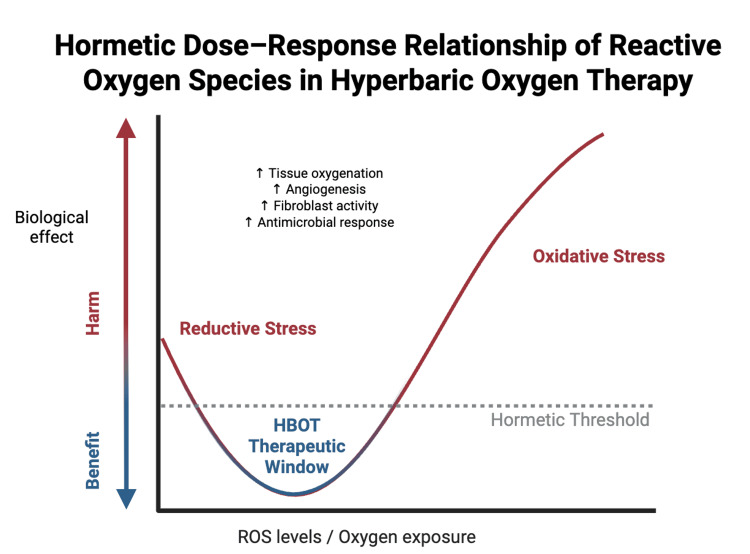
Hormetic dose-response relationship of reactive oxygen species (ROS) in hyperbaric oxygen therapy Moderate increases in ROS, generated during HBOT, trigger adaptive cellular responses that enhance tissue oxygenation, angiogenesis, fibroblast activity, and antimicrobial defense. This hormetic therapeutic window promotes tissue repair and wound healing, whereas excessive ROS production may lead to oxidative stress and potential cellular injury. This image is an original author-created schematic using Biorender (BioRender Inc., Toronto, Canada) and was not generated using AI. HBOT: hyperbaric oxygen therapy

Experimental and translational studies continue to expand the mechanistic understanding of HBOT. Animal wound models have demonstrated synergistic effects when hyperbaric oxygen is combined with adjunct therapies, such as platelet-rich plasma, extracorporeal shockwave therapy, and advanced cellular matrices, suggesting a multimodal regenerative role [4,9-11]. At the molecular level, HBOT-induced increases in ROS can trigger adaptive cellular responses that promote angiogenesis, fibroblast proliferation, and antimicrobial activity when maintained within a therapeutic hormetic range. As illustrated in Figure 2, this hormetic dose-response relationship highlights how controlled ROS generation may stimulate beneficial regenerative pathways, whereas excessive oxidative stress may lead to cellular injury. Collectively, these molecular, cellular, and microvascular mechanisms establish HBOT as a biologically active intervention capable of enhancing wound healing across multiple physiological levels.

Clinical Applications in Chronic and Ischemic Surgical Wounds

The clinical application of HBOT in wound management has been most extensively explored in chronic and ischemic surgical wounds, where impaired perfusion and sustained hypoxia significantly hinder physiological tissue repair. Among these, diabetic foot ulcers represent one of the most studied indications for adjunctive HBOT. Multiple systematic reviews and meta-analyses have demonstrated that HBOT improves wound closure rates, accelerates epithelialization, and reduces the incidence of major amputations when incorporated into multidisciplinary treatment protocols [2,9,11]. In addition to enhancing tissue oxygenation, HBOT has been shown to potentiate host immune responses and augment antibiotic efficacy, both of which are critical in infection-prone diabetic wounds.

Chronic venous ulcers constitute another clinical domain in which HBOT has demonstrated therapeutic benefit. Persistent venous hypertension leads to microcirculatory dysfunction, leukocyte trapping, and progressive tissue hypoxia, ultimately resulting in refractory wounds resistant to conventional compression therapy. Adjunctive hyperbaric oxygen has been associated with improved granulation tissue formation, enhanced epithelial migration, and reduced ulcer surface area in patients with recalcitrant venous disease [10,12,13]. These findings support the incorporation of HBOT as a complementary modality in advanced wound care settings.

Burn injuries represent an additional area of clinical application. Thermal trauma induces microvascular thrombosis, edema formation, and compromised oxygen delivery within the zone of stasis. HBOT mitigates burn wound progression through vasoconstriction-mediated edema reduction, while simultaneously increasing oxygen availability to ischemic tissues. Clinical and experimental evidence suggests that HBOT may improve graft take, reduce infection rates, and shorten healing intervals in complex burn wounds [14-16].

Radiation-induced soft tissue injury remains one of the most established surgical indications for hyperbaric therapy. Chronic radiation wounds are characterized by fibrosis, obliterative endarteritis, and severe hypoxia, creating an inhospitable environment for surgical reconstruction. Evidence from controlled trials and systematic reviews indicates that HBOT enhances angiogenesis, improves tissue elasticity, and facilitates surgical wound closure in irradiated fields [1]. The role of hyperbaric oxygen in this setting has become particularly relevant in reconstructive procedures, where vascular compromise limits healing potential.

Beyond chronic ulcerative and radiation-related wounds, HBOT has been utilized in complex postoperative surgical complications, including wound dehiscence, necrotizing infections, and ischemic soft tissue defects. Clinical reports highlight improved infection control, enhanced tissue viability, and reduced progression of necrosis following adjunctive hyperbaric therapy [17,18]. These applications underscore the versatility of HBOT across diverse surgical wound etiologies.

Collectively, the expanding body of clinical evidence supports HBOT as a valuable adjunct in the management of chronic and ischemic surgical wounds. Its ability to simultaneously enhance perfusion, modulate inflammation, and potentiate antimicrobial defenses positions HBOT as an integrative tool within modern, multidisciplinary wound care frameworks. A summary of the principal clinical evidence supporting the use of HBOT in chronic and ischemic surgical wounds is presented in Table 1.

HBOT in Surgical Tissue Salvage

HBOT has gained increasing relevance in the context of surgical tissue salvage, particularly in clinical scenarios characterized by critical ischemia, compromised perfusion, and impending reconstructive failure. The success of reconstructive procedures, such as skin grafts, local flaps, and free tissue transfers, depends heavily on adequate oxygen delivery during the early postoperative period. When vascular compromise occurs, tissue hypoxia rapidly precipitates necrosis, infection, and reconstructive loss. Adjunctive HBOT has therefore emerged as a valuable supportive modality, aimed at restoring tissue oxygenation and improving salvage outcomes.

One of the principal mechanisms through which HBOT contributes to tissue salvage is the enhancement of microvascular perfusion within ischemic flaps and grafts. Hyperoxia promotes capillary angiogenesis, reduces interstitial edema through vasoconstriction, and improves oxygen diffusion across compromised vascular territories. These physiological effects are particularly relevant in the setting of ischemia-reperfusion injury, where oxidative stress and endothelial dysfunction threaten tissue viability following surgical revascularization [7,18,19].

Clinical evidence supporting the use of HBOT in reconstructive salvage continues to expand. Reports across trauma and limb reconstruction literature have demonstrated improved graft integration, reduced necrosis progression, and enhanced wound stability when hyperbaric therapy is incorporated into multidisciplinary salvage protocols [7]. Additionally, HBOT has shown benefit in crush injuries and complex soft tissue trauma, where microcirculatory collapse impairs physiological healing capacity [18,19].

The role of hyperbaric oxygen in complex wound reconstruction extends beyond extremity trauma. Necrotizing infections, ischemic postoperative wounds, and graft-compromised defects have all been described as clinical scenarios where adjunctive HBOT may reduce tissue loss and improve reconstructive readiness. Observational studies have reported enhanced infection control, improved granulation tissue formation, and higher rates of successful secondary closure following hyperbaric therapy [18,20].

In recent years, growing attention has been directed toward the application of HBOT within aesthetic and reconstructive surgical practice. A systematic review and meta-analysis evaluating HBOT as an adjunct in aesthetic surgery demonstrated improved postoperative healing outcomes, reduced complication rates, and enhanced tissue recovery across multiple cosmetic procedures [21]. These findings suggest that the regenerative and antimicrobial properties of HBOT may extend beyond traditional wound care into elective surgical optimization.

Complementing these observations, broader narrative syntheses examining HBOT across surgical specialties have reinforced its role as a mechanistically active adjunct in tissue salvage. By integrating molecular, microvascular, and immunomodulatory pathways, HBOT contributes to graft survival, flap viability, and reconstructive success across diverse surgical domains [22]. Such integrative perspectives underscore the expanding therapeutic scope of hyperbaric medicine within contemporary surgical practice.

Breast and soft tissue reconstructive procedures represent additional areas where HBOT has demonstrated salvage potential. In the setting of ischemic mastectomy flaps or compromised nipple-sparing reconstructions, HBOT has been associated with improved tissue perfusion and reduced necrosis rates, supporting its adjunctive role in breast reconstruction salvage algorithms [23-25].

Collectively, the available evidence highlights HBOT as a valuable adjunct in surgical tissue salvage. Its capacity to enhance oxygen delivery, promote angiogenesis, modulate inflammation, and improve antimicrobial defense positions HBOT as a supportive modality capable of optimizing reconstructive outcomes in high-risk surgical scenarios. Key clinical evidence supporting the role of HBOT in reconstructive tissue salvage is summarized in Table 2.

Clinical Outcomes

HBOT has demonstrated measurable benefits across multiple clinical endpoints in surgical wound management and reconstructive care. Among the most consistently reported outcomes is the acceleration of wound healing. By enhancing tissue oxygenation and promoting angiogenesis, HBOT facilitates faster epithelialization, improved collagen matrix deposition, and more rapid progression through the proliferative phase of healing. Clinical studies evaluating chronic wound populations have reported shorter times to wound closure and higher rates of complete epithelialization when hyperbaric oxygen is incorporated into standard wound care protocols [2,9,11].

Complication Reduction

In addition to expediting tissue repair, HBOT has been associated with significant reductions in postoperative complication rates. Surgical site infections, particularly in ischemic or contaminated wounds, represent a major contributor to morbidity and reconstructive failure. Hyperoxia enhances leukocyte bactericidal activity and improves antibiotic penetration into poorly perfused tissues, thereby strengthening host defense mechanisms. Clinical observations across chronic wounds, burn injuries, and reconstructive procedures have demonstrated lower infection rates in patients receiving adjunctive hyperbaric therapy [8,14,15].

Necrosis prevention and tissue preservation constitute another critical outcome domain. In compromised grafts and flaps, insufficient oxygen delivery often precipitates progressive ischemia and tissue loss. HBOT mitigates this process by improving microvascular perfusion, reducing edema, and supporting angiogenic recovery. Studies in reconstructive trauma, breast surgery, and complex postoperative wounds have reported reduced necrosis progression and higher rates of successful tissue salvage following HBOT administration [21,23,24].

Amputation risk reduction represents one of the most clinically impactful benefits reported in the literature, particularly in diabetic and ischemic wound populations. Systematic reviews and meta-analyses have shown that adjunctive HBOT significantly lowers the likelihood of major limb amputation by promoting wound closure and controlling infection [2,5].

From a safety perspective, HBOT is generally well tolerated when administered within established pressure and duration parameters. Reported adverse events are uncommon and typically mild, including barotrauma-related otic discomfort or transient visual changes. Serious complications, such as oxygen toxicity and seizures, remain rare when standard therapeutic protocols are followed [22].

Cost-effectiveness analyses, though limited, suggest that the upfront resource utilization associated with HBOT may be offset by reductions in reoperation rates, hospitalization duration, and long-term disability associated with wound complications and amputations. As such, HBOT may represent not only a clinically effective adjunct but also a resource-efficient strategy in high-risk surgical populations.

Collectively, the available evidence underscores the multifaceted clinical impact of HBOT. Through its capacity to accelerate wound healing, reduce infection and necrosis, preserve tissue viability, and improve reconstructive outcomes, HBOT continues to solidify its role as a valuable adjunct within modern surgical care pathways.

Discussion

The present narrative review synthesizes current evidence regarding the mechanistic foundations and clinical applications of HBOT in surgical wound healing and tissue salvage. The available literature consistently supports HBOT as a biologically active adjunct, capable of modulating tissue hypoxia, enhancing microvascular perfusion, and improving reconstructive outcomes across diverse surgical contexts. However, the expanding scope of hyperbaric medicine warrants a critical appraisal of its translational applicability, clinical limitations, and future integration into multidisciplinary surgical care.

One of the most compelling aspects of HBOT is its versatility across surgical subspecialties. Although traditionally associated with chronic wound care and radiation injury, contemporary applications now extend into gynecologic oncology, reconstructive urology, and complex abdominal wound management. Clinical guidance frameworks have highlighted the utility of HBOT in managing irradiated pelvic tissues, postoperative dehiscence, and ischemic gynecologic surgical wounds, reinforcing its role beyond conventional wound paradigms [26]. Similarly, case series within reconstructive urology have demonstrated improved healing and reduced complication rates in complex genitourinary wound defects following adjunctive hyperbaric therapy [27].

Beyond reconstructive salvage, emerging literature has explored the role of HBOT within aesthetic and elective surgical settings. Systematic reviews evaluating HBOT in aesthetic medicine and anti-aging applications suggest potential benefits in tissue regeneration, postoperative recovery, and dermal remodeling [28]. Complementary clinical investigations in facial aesthetic surgery have reported improved wound healing, reduced edema, and enhanced scar quality in patients receiving adjunctive hyperbaric therapy following rhytidectomy procedures [29]. These findings support the concept that HBOT may extend beyond salvage therapy into perioperative optimization strategies within elective surgical practice.

Scar modulation represents another evolving application. Prospective comparative studies have suggested that HBOT may influence collagen organization and fibroblast activity, potentially improving postoperative scar outcomes when compared with standard interventions, such as silicone sheeting [30]. While preliminary, these findings raise important considerations regarding the broader regenerative implications of hyperbaric therapy within surgical wound remodeling phases.

Predictive modeling and outcome stratification have also begun to emerge within hyperbaric research. Decision tree-based analytical approaches have been proposed to identify clinical variables associated with HBOT success, including wound chronicity, perfusion status, and comorbidity burden [31]. Such predictive tools may facilitate more precise patient selection, optimizing resource allocation and therapeutic efficacy within hyperbaric programs.

From a multidisciplinary perspective, the integration of HBOT with adjunctive technologies represents a promising frontier. Randomized and protocol-driven investigations combining hyperbaric oxygen with endovascular revascularization strategies for chronic limb-threatening ischemia aim to synergistically address both macrovascular and microvascular perfusion deficits [32]. Similarly, combination approaches integrating HBOT with negative pressure wound therapy have demonstrated improved healing dynamics in chronic wound populations, suggesting additive regenerative benefits [32,33].

The systemic therapeutic reach of HBOT is further illustrated in inflammatory and gastrointestinal wound contexts. Evidence from observational studies and case series has documented successful application of HBOT in refractory Crohn’s disease-associated fistulizing wounds, where enhanced tissue oxygenation may support mucosal repair and infection control [34,35]. Although outside traditional surgical wound paradigms, these findings - primarily derived from lower-level evidence - underscore the broader regenerative and immunomodulatory capacity of hyperbaric therapy.

Despite these expanding indications, several limitations must be acknowledged. The current body of evidence supporting HBOT in surgical wound healing remains predominantly composed of observational studies, case series, and small cohort analyses, with considerable heterogeneity in treatment protocols, pressure regimens, and outcome reporting. High-quality randomized controlled trials are relatively scarce, limiting the strength of causal inferences and the generalizability of findings.

Furthermore, variability in patient selection, timing of intervention, and adjunctive therapies introduces additional methodological complexity. While emerging multicenter investigations may provide higher-level evidence in the future, the present literature base necessitates cautious interpretation of reported benefits. Accessibility, cost, and infrastructure requirements continue to constrain widespread HBOT implementation, particularly in resource-limited settings.

Future research directions should prioritize standardized treatment protocols, robust comparative trials, and cost-effectiveness analyses. Integration of molecular biomarkers and predictive analytics may further refine patient selection and therapeutic personalization. As regenerative medicine and reconstructive surgery continue to evolve, HBOT is poised to assume an increasingly integrative role within perioperative optimization and tissue salvage frameworks.

## Conclusions

HBOT has emerged as a biologically active and clinically relevant adjunct in contemporary surgical wound management and tissue salvage. By reversing tissue hypoxia and enhancing angiogenesis, inflammatory modulation, and antimicrobial activity, HBOT contributes to multiple physiological processes involved in tissue repair and regeneration. The available clinical literature suggests potential benefits across several surgical contexts, including chronic ischemic wounds, compromised grafts and flaps, and complex postoperative complications. These effects may translate into improved wound closure, enhanced tissue viability, and reduced risk of infection and necrosis in selected, high-risk patient populations.

Despite these promising findings, the current evidence base remains heterogeneous, with variability in study design, treatment protocols, and patient selection. Future research should prioritize standardized therapeutic algorithms, well-designed comparative trials, and cost-effectiveness analyses to better define the role of HBOT in modern surgical care. As regenerative medicine and multidisciplinary wound management strategies continue to evolve, HBOT may play an increasingly important role as a complementary modality in optimizing complex wound healing and reconstructive outcomes.

## References

[1] Lin ZC, Bennett MH, Hawkins GC, Azzopardi CP, Feldmeier J, Smee R, Milross C (2023). Hyperbaric oxygen therapy for late radiation tissue injury. Cochrane Database Syst Rev.

[2] Sharma R, Sharma SK, Mudgal SK, Jelly P, Thakur K (2021). Efficacy of hyperbaric oxygen therapy for diabetic foot ulcer, a systematic review and meta-analysis of controlled clinical trials. Sci Rep.

[3] Oropallo AR, Serena TE, Armstrong DG, Niederauer MQ (2021). Molecular biomarkers of oxygen therapy in patients with diabetic foot ulcers. Biomolecules.

[4] Chen RF, Lin YN, Liu KF (2023). Compare the effectiveness of extracorporeal shockwave and hyperbaric oxygen therapy on enhancing wound healing in a streptozotocin-induced diabetic rodent model. Kaohsiung J Med Sci.

[5] Zhang Z, Zhang W, Xu Y, Liu D (2022). Efficacy of hyperbaric oxygen therapy for diabetic foot ulcers: an updated systematic review and meta-analysis. Asian J Surg.

[6] Keohane C, Westby D, Nolan FC, Twyford M, Tawfick W, Walsh SR (2023). Hyperbaric oxygen as an adjunct in the treatment of venous ulcers: a systematic review. Vasc Endovascular Surg.

[7] Marra C, Pentangelo P, Losco L, Ceccaroni A, Barbato A, Alfano C (2024). Lower extremity trauma: a multidimensional reconstructive approach with hyperbaric oxygen therapy. J Clin Med.

[8] Zhou D, Fu D, Yan L, Xie L (2023). The role of hyperbaric oxygen therapy in the treatment of surgical site infections: a narrative review. Medicina (Kaunas).

[9] Seyhan N, Öksüz S (2022). Effect of hyperbaric oxygen therapy when combined with fresh and frozen platelet-rich plasma on chronic wound healing in rats. Ulus Travma Acil Cerrahi Derg.

[10] Popescu V, Cauni V, Petrutescu MS (2023). Chronic wound management: from gauze to homologous cellular matrix. Biomedicines.

[11] Hu X, Meng H, Liang J (2025). Comparison of the efficacy of 12 interventions in the treatment of diabetic foot ulcers: a network meta-analysis. PeerJ.

[12] Lalieu RC, Akkerman I, van Hulst RA (2021). Hyperbaric oxygen therapy for venous leg ulcers: a 6 year retrospective study of results of a single center. Front Med (Lausanne).

[13] Elsharnoby AM, El-Barbary AH, Eldeeb AE, Hassan HA (2025). Resistant chronic venous leg ulcers: effect of adjuvant systemic hyperbaric oxygen therapy versus venous intervention alone. Int J Low Extrem Wounds.

[14] Weitgasser L, Ihra G, Schäfer B, Markstaller K, Radtke C (2021). Update on hyperbaric oxygen therapy in burn treatment. Wien Klin Wochenschr.

[15] Oley MH, Oley MC, Aling DM (2021). Effects of hyperbaric oxygen therapy on the healing of thermal burns and its relationship with ICAM-1: a case-control study. Ann Med Surg (Lond).

[16] Oley MH, Oley MC, Wewengkang LA (2022). Bactericidal effect of hyperbaric oxygen therapy in burn injuries. Ann Med Surg (Lond).

[17] Teguh DN, Bol Raap R, Koole A, Knippenberg B, Smit C, Oomen J, van Hulst RA (2021). Hyperbaric oxygen therapy for nonhealing wounds: treatment results of a single center. Wound Repair Regen.

[18] Zaman T, Canarslan Demir K, Gunduz SH, Gulap Y, Basak AM, Yilmaz KB (2025). Hyperbaric oxygen therapy as an effective adjunctive treatment in the reconstruction of tissue defects with graft in diabetic foot patients: a retrospective cohort study. Int Wound J.

[19] Chang DH, Hsieh CY, Chang CW, Wang HH, Chang HT (2024). The use of hyperbaric oxygen therapy in the treatment of hand crush injuries. Wound Repair Regen.

[20] Feres O, Feitosa MR, Ribeiro da Rocha JJ (2021). Hyperbaric oxygen therapy decreases mortality due to Fournier's gangrene: a retrospective comparative study. Med Gas Res.

[21] Mortada H, González JE, Husseiny YM, Al Jabbar I, Sultan F, Alrobaiea S, Neel OF (2025). Efficacy of hyperbaric oxygen therapy as an adjunct in aesthetic surgery: a systematic review and meta-analysis of postoperative outcomes and complications. Aesthetic Plast Surg.

[22] González Flores JE, Vázquez Hernández DB, Gonzalez Espinosa A, Sandoval Polito A, Navalón Calzada A, Romero Cázares EO (2026). Hyperbaric oxygen therapy in modern surgical practice: mechanistic basis and clinical applications across specialties. Cureus.

[23] Nasr HY, Rifkin WJ, Muller JN, Chiu ES (2023). Hyperbaric oxygen therapy for threatened nipple-sparing mastectomy flaps: an adjunct for flap salvage. Ann Plast Surg.

[24] Idris OA, Ahmedfiqi YO, Shebrain A (2024). Hyperbaric oxygen therapy for complications in nipple-sparing mastectomy with breast reconstruction: a systematic review. J Clin Med.

[25] Hanna MK (2021). The contribution of preconditioning hyperbaric oxygen for complex re-operative surgery of bladder exstrophy and epispadias. A case study of 11 patients. J Pediatr Urol.

[26] Foy OB, Kumar A, Liang MI (2025). Hyperbaric oxygen therapy: a practical guide for gynecologic oncologists. Gynecol Oncol Rep.

[27] Oley MH, Oley MC, Iskandar AA, Toreh C, Tulong MT, Faruk M (2021). Hyperbaric oxygen therapy for reconstructive urology wounds: a case series. Res Rep Urol.

[28] Fisher SM, Sherif RD, Borab ZM, Ganesh Kumar N, Rohrich RJ (2025). Hyperbaric oxygen therapy in aesthetic medicine and anti-aging: a systematic review. Aesthetic Plast Surg.

[29] Neel OF, Mousa AH, Al-Terkawi RA, Bakr MM, Mortada H (2023). Assessing the efficacy of hyperbaric oxygen therapy on facelift outcomes: a case-control study comparing outcomes in patients with and without hyperbaric oxygen therapy. Aesthet Surg J Open Forum.

[30] Chen PC, Liao TC, Chou CY, Chu CM, Hsiao PJ, Tsai HC (2025). Comparative efficacy of silicone sheets and hyperbaric oxygen therapy in post-surgical scar prevention: a prospective observational study. Int J Med Sci.

[31] Oley MH, Oley MC, Langi FL (2021). Predicting hyperbaric oxygen therapy success using the decision tree approach. Ann Med Surg (Lond).

[32] Sato Y, Urasawa K, Morishita T (2021). Combined treatment with hyperbaric oxygen therapy and endovascular therapy for patients with chronic limb-threatening ischemia - study protocol for the HOTFOOT multicenter randomized controlled trial. Circ Rep.

[33] Riansrithongkham T, Yongchareon P, Sivadechathep A, Likitvong A, Mahamongkol T, Pruksapong C (2025). Efficacy of hyperbaric oxygen therapy combine with negative pressure wound therapy in chronic wound: a randomized controlled trial. JPRAS Open.

[34] Feitosa MR, Parra RS, Machado VF (2021). Adjunctive hyperbaric oxygen therapy in refractory Crohn's disease: an observational study. Gastroenterol Res Pract.

[35] Leong JW, Yan ZH, Foo FJ, Koh FH, Cheng LT, Kong SC, Tey TT (2024). Hyperbaric oxygen therapy achieved fistula healing in a young patient with severe refractory perianal Crohn's disease. Cureus.

